# Analysis of ultrasound parameters influencing endometrial receptivity and a pregnancy outcomes predictive model for patients undergoing *in vitro* fertilization and embryo transfer: a prospective study

**DOI:** 10.3389/fendo.2025.1677593

**Published:** 2025-11-18

**Authors:** Chunlian Wang, Jiao Lin, Xingping Zhao, Ni Liu, Pengzi Sun, Jiaoli Yang, Xue Zhou

**Affiliations:** 1 Obstetrics and Gynecology Ultrasound Department, Xiangtan Central Hospital, Affiliated Hospital of Hunan University, Xiangtan, Hunan, China; 2 Reproductive Center, Xiangtan Central Hospital, Affiliated Hospital of Hunan University, Xiangtan, Hunan, China; 3 Reproductive Center, Third Xiangya Hospital, Affiliated Hospital of Zhongnan University, Changsha, Hunan, China

**Keywords:** ultrasound parameters, endometrial contrast-enhanced ultrasound, IVF-ET, ongoing pregnancy, prediction model

## Abstract

**Purpose:**

This study aims to assess the impact of ultrasound parameters on endometrial receptivity in patients undergoing IVF-ET and to establish a predictive model for ongoing pregnancy outcomes.

**Methods:**

The prospective cohort study included 86 patients treated at the Reproductive Center of Xiangtan Central Hospital from May to December 2024. Participants underwent multimodal ultrasound evaluation one day before embryo transfer. The study analyzed endometrial morphology, blood flow parameters, as well as threedimensional power Doppler angiography(3D-PDA), and endometrial contrast-enhanced ultrasound (CEUS) indicators. Multimodal ultrasound parameters were used to establish a predictive model for sustained pregnancy.

**Results:**

Among the 86 patients, 42 (48.8%) achieved ongoing pregnancy, while 44 (51.2%) did not. Significant differences between the groups were observed in the number of mature oocytes and endometrial blood flow grading (both P = 0.005). Lasso regression identified eight predictive variables: primary cause of infertility, baseline luteinizing hormone (LH) levels, number of MII oocytes, uterine cavity volume, endometrial blood flow grading, subendometrial flow index (FI) in 3D-PDA, and endometrial and subendometrial peak intensity (PI) in CEUS. The aforementioned variables as well as embryonic factors were integrated into eight machine learning models, with the Gradient Boosting model exhibiting superior predictive performance (AUC: 0.981). SHapley Additive exPlanations (SHAP) analysis indicated that a higher number of MII oocytes, improved endometrial blood flow, specific infertility etiologies, elevated baseline LH levels, and reduced subendometrial/endometrial PI, subendometrial FI, and uterine cavity volume were associated with a greater likelihood of pregnancy.

**Conclusion:**

The integration of 3D-PDA and CEUS technologies shifts IVF-ET evaluation from traditional morphological observation to functional assessment, offering a new perspective for predicting sustained pregnancy outcomes. This innovation shows promising clinical potential by optimizing treatment strategies like MII oocyte retrieval, improving endometrial blood flow grading, and adjusting blood flow parameters (PI and FI), significantly enhancing pregnancy success rates and advancing assisted reproductive technologies.

## 1 Introduction

Endometrial receptivity (ER) represents a transient yet critical endometrial state that facilitates blastocyst apposition, adhesion, and invasion, while concurrently promoting stromal remodeling to enable successful embryo implantation (1). As a pivotal determinant of pregnancy success in IVF-ET cycles, optimal ER necessitates synchronized endometrial thickening and vascularization. Endometrial arterial perfusion and the development of a robust vascular network serve as essential biomarkers for predicting pregnancy outcomes. Doppler ultrasound evaluations of uterine blood flow parameters reveal significant correlations with implantation potential, demonstrating consistently lower resistance index (RI) and pulsatility index (PI) values in endometrial blood flow among pregnant women compared to their non-pregnant counterparts (2). Elevated PI and RI values indicate increased vascular resistance, leading to compromised endometrial perfusion and, consequently, reduced receptivity. Ultrasonographic assessments of endometrial blood flow have been associated with embryo transfer success, with studies reporting higher subendometrial blood flow parameters—vascularization index (VI), flow index (FI), and vascularization-flow index (VFI)—in pregnant groups (3–5). However, traditional Doppler indices of spiral arteries (RI, PI, peak systolic velocity [PSV]) exhibit limited predictive value for pregnancy outcomes. Research by Maged et al. (6) and others found no significant differences in VI and FI between conception and non-conception cycles, while logistic regression analyses failed to establish meaningful associations between pregnancy outcomes and various hemodynamic parameters (7). These conflicting findings underscore persistent controversies regarding their clinical utility.

Given the inadequacy of single indicators in comprehensively assessing endometrial receptivity, research has increasingly shifted toward multimodal ultrasound evaluations. Jiao et al. (8) developed a scoring system to predict early miscarriage, incorporating parameters such as endometrial thickness (EMT), morphology, peristalsis, volume, and blood flow. Their results demonstrated significantly lower scores in the miscarriage group (10.46 ± 2.99) compared to the successful pregnancy group (13.49 ± 2.21). Similarly, Liao et al. (9) proposed a model integrating EMT, volume, and vascular blood flow indices, identifying a multimodal ultrasound endometrial score below 12 as a risk factor for compromised full-term delivery post-transfer. Li et al. (10) further advanced this field by employing clinical indicators—including EMT, PI, RI, and ultrasound elastography—to develop a logistic model with 76.92% predictive accuracy for pregnancy outcomes. Despite these advancements, current ultrasound-based scoring systems remain insufficient for reliably predicting IVF-ET success.

An optimal blood supply to the endometrium is essential for embryo implantation. During early implantation, endometrial vascular permeability increases, causing significant changes in the microvasculature of endometrium and subendometrial region. As the primary decidual zone develops, capillaries near the embryo close while those adjacent to the decidual zone dilate. Impaired endometrial angiogenesis may lead to recurrent implantation failure andpregnancy loss. Increased vascular permeability aids in delivering growth factors and cytokines to the implantation site (11). CEUS provides real-time visualization of tissue microcirculation, while 3D-PDA effectively detects low-velocity blood flow with outangle limitations. 3D-PDA captures blood flow signals from all directions, enabling comprehensive volume analysis through computer reconstruction. This study seeks to address this gap by developing a more precise evaluation model that incorporates a comprehensive array of ultrasound indicators related to ER, encompassing physiological and morphological characteristics, blood flow dynamics, and advanced imaging parameters such as 3D-PDA and CEUS.

## 2 Method

### 2.1 Study population and data collection

This prospective cohort study was conducted at the Reproductive Center of Xiangtan Central Hospital from May to December 2024, enrolling 86 consecutive patients undergoing IVF-ET treatment. A standardized protocol was implemented for comprehensive data collection, including detailed assessment of: (1) endometrial morphological characteristics (thickness, trilaminar pattern, and volumetric measurements); (2) functional parameters (elasticity measurements and peristaltic activity); (3) hemodynamic evaluations through Doppler assessment of endometrial and subendometrial blood flow; (4) three-dimensional power Doppler angiography (3D-PDA) quantification of vascular indices (FI, VI, VFI); and (5) endometrial contrast-enhanced ultrasound (CEUS) parameters (AT, TTP, RT, PI, AUC).

Eligibility Criteria: Participants were selected based on the following inclusion criteria: (i) documented infertility with medical indication for IVF-ET, including tubal factor, anovulatory disorders resistant to medical therapy, stage I-II endometriosis, severe male factor infertility (total motile sperm count <5×10^6^), or unexplained infertility; (ii) age <40 years with availability of ≥1 high-quality blastocyst for transfer; (iii) willingness to provide informed consent. Exclusion criteria comprised: (i) structural uterine abnormalities (Asherman’s syndrome, submucosal fibroids, endometrial polyps); (ii) active systemic or psychiatric comorbidities; (iii) substance abuse disorders; (iv) recent exposure to teratogens or gonadotoxic agents; (v) known contrast media hypersensitivity; (vi) autoimmune conditions requiring biologic therapies.

The final cohort of 86 participants was established after rigorous screening and verification of eligibility criteria. The study protocol was approved by the Institutional Ethics Committee of Xiangtan Central Hospital (Approval No. SZ202211-05) and conducted in accordance with Good Clinical Practice guidelines. All participants provided written informed consent after detailed counseling about study procedures.

### 2.2 Multimodal ultrasound assessment

Ultrasound was performed by one senior ultrasonographer on the day of the ET. Standardized two-dimensional transvaginal ultrasound was performed to obtain median sagittal uterine views for endometrial assessment. Endometrial thickness was measured at its maximal dimension perpendicular to the uterine cavity. Endometrial morphology was classified according to Gonen criteria: Type A demonstrating a distinct trilaminar pattern with hyperechoic outer lines and hypoechoic central cavity; Type B showing intermediate echogenicity with partial loss of trilaminar appearance; Type C characterized by homogeneous hyperechogenicity without visible layering. Endometrial peristaltic waves were observed for two minutes under stable conditions, with data analyzed at quadruple speed to assess type, direction, frequency, and intensity. The waves were categorized into five types: forward, reverse, static, bidirectional, and localized. Doppler evaluation was conducted using standardized protocols. Endometrial and subendometrial vascular patterns were classified per Applebaum criteria: Type I (peripheral vascularity limited to the hypoechoic junctional zone); Type II (vascular penetration through the hyperechoic endometrial border); Type III (intraendometrial vascularization).

3D PDA and CEUS examinations were conducted with Mindray color ultrasound system(Nuewa R9Q), Switch to three-dimensional power Doppler mode, Using an intracavitary volume probe (Model DE10-3WU), 3D-PDA images of the endometrium were captured with preset scanning parameters: 120° angle, 0.9 kHz PRF, 71 Hz filter, and 40 gain. The system’s Smart ERA function automatically reconstructed the endometrium in 3D and calculated volume and blood flow parameters (VI, FI, VFI). Enable Shell functionality, set the regions of interest (ROIs) from the endometrial fundus to above the internal cervical os, including the subendometrial area within 3 mm of the endometrial-myometrial junction.

The patient followed a standard protocol during the CEUS exam. Standardized settings: MI between 0.065-0.099, transducer frequency at CH4-CH5 MHz, dynamic range at 100 dB, iClear at 1, pseudocolor at 5, smoothing at 1, and gain at 43. The procedure involved obtaining a midsagittal section of the uterus, switching to contrast mode, and adjusting the image for clear endometrial visibility. After injecting 2.4 ml of SonoVue and 10 ml of saline, the timer was started to record contrast time. Images were captured for 120 seconds, and the patient was monitored for 30 minutes post-exam to ensure no discomfort. During image analysis, real-time monitoring of contrast agent perfusion in the endometrium was conducted, and time-intensity curves (TIC) were generated. The selection of ROI is consistent with 3D-PDA. The ultrasound machine’s TIC analysis software automatically plotted the curves, from which quantitative parameters such as PI, AUC, TTP, AT were derived. An experienced physician performed and plotted TIC curves for all patients, averaging three tracings for each quantitative parameter.

### 2.3 Reproductive outcomes

IVF treatment outcomes were evaluated through serial serum β-human chorionic gonadotropin (β-hCG) measurements, with the initial quantitative assessment performed 12 days post-embryo transfer. A positive biochemical pregnancy was defined as β-hCG ≥50 IU/L, followed by serial monitoring to confirm appropriate doubling kinetics. Clinical pregnancy confirmation required transvaginal ultrasound visualization of an intrauterine gestational sac with detectable cardiac activity at 6 weeks’ gestation. Ongoing pregnancy was defined as the presence of a viable fetus confirmed by ultrasound beyond 14 weeks’ gestation. For analytical purposes, outcomes were dichotomized: “Yes” indicated confirmed ongoing intrauterine pregnancy at 14 weeks, while “No” encompassed negative results, biochemical pregnancies (isolated β-hCG elevation without clinical confirmation), or early pregnancy loss prior to 14 weeks.

### 2.4 Statistical methods

Analyses were conducted using R v4.4.1 and Python v3.12.0. Continuous and categorical variables were reported as mean ± SD and frequencies (%), respectively. Group comparisons used χ² tests for categorical variables and Mann-Whitney U tests for continuous variables. Predictor selection employed LASSO regression (10-fold CV, λ=0.576), identifying eight key variables: infertility etiology, baseline LH, MII oocyte count, uterine volume, endometrial blood flow grade, subendometrial FI (3D-PDA), endometrial/subendometrial PI (CEUS). These predictors were evaluated in multiple machine learning models, with optimal model selection based on AUC performance. Final model interpretation used SHAP analysis.

## 3 Result

### 3.1 Factors associated with ongoing pregnancy

The study cohort comprised 86 patients, of whom 42 (48.8%) achieved ongoing pregnancy, while 44 (51.2%) did not (including 30 non-pregnant cases, 10 biochemical pregnancies, and 2 early miscarriages). Significant differences were observed in the number of MII oocytes and endometrial blood flow grading (both P = 0.005)(**Table 1**). No statistically significant differences were found in baseline characteristics, endometrial morphology/physiology, or 3D-PDA and CEUS parameters (P > 0.05) (**Table 2**).

**Table 1:** Comparison of baseline parameters between the two groups.

Variables	Ongoing pregnancy		Statistic	*P*
	No (n = 44)	Yes (n = 42)		
Age	33.59 ± 5.04	32.79 ± 3.75	-0.47	0.640
AMH	3.13 ± 1.98	4.38 ± 3.97	-1.12	0.263
BMI	23.11 ± 3.64	22.87 ± 3.11	-0.32	0.749
Infertility years	3.41 ± 3.40	3.11 ± 2.41	-0.07	0.944
Baseline FSH	6.97 ± 1.60	6.77 ± 1.74	-0.14	0.887
Baseline LH	4.91 ± 1.53	6.05 ± 3.18	-1.38	0.167
Infertility factors, n(%)			–	0.200
Female factor	39 (88.64)	30 (71.43)		
Male factor	3 (6.82)	5 (11.90)		
RSA	1 (2.27)	5 (11.90)		
Combined factors	1 (2.27)	2 (4.76)		
Ovulation induction protocol, n(%)			2.00	0.368
Antagonist protocol	22 (50.00)	25 (59.52)		
Short protocol	13 (29.55)	7 (16.67)		
Long protocol in the early follicular phase	9 (20.45)	10 (23.81)		
MII oocyte number	8.59 ± 4.07	11.62 ± 5.40	-2.81	0.005**
CD138, n(%)			0.29	0.866
(-)	16 (36.36)	16 (38.10)		
(+)	21 (47.73)	21 (50.00)		
unknow	7 (15.91)	5 (11.90)		
Inflammation reduction cycle	2.40 ± 1.20	2.15 ± 0.99	-1.06	0.289
Endometrial preparation protocol, n(%)			–	0.545
GnRHa_HRT	22 (50.00)	24 (57.14)		
HRT	20 (45.45)	18 (42.86)		
NC	2 (4.55)	0 (0.00)		
Number of high-quality embryos				
0	18 (40.91)	10 (23.81)	3.55	0.169
1	16 (36.36)	23 (54.76)		
2	10 (22.73)	9 (21.43)		

AMH, Anti-Müllerian hormone; BMI, Body Mass Index; HRT, hormone replacement therapy; NC, Natural Cycles; RSA, Recurrent Spontaneous Abortion; -, Fisher exact. **P<0.01.

**Table 2:** Comparison of ultrasound parameters between the two groups.

Variables	Ongoing pregnancy		Statistic	*P*
	No (n = 44)	Yes (n = 42)		
EMT on ultrasound day(mm)	9.16 ± 2.21	9.12 ± 1.52	-0.06	0.955
EV on ultrasound day(cm^3^)	4.22 ± 2.26	3.67 ± 1.16	-0.52	0.604
Endometrial peristalsis	3.52 ± 2.20	4.26 ± 2.16	-1.45	0.147
Endometrial 3D-PDA blood flow				
VI	10.55 ± 10.88	8.90 ± 8.19	-0.38	0.707
FI	23.40 ± 15.87	26.39 ± 30.70	0.00	1.000
VFI	2.15 ± 2.28	1.81 ± 1.91	-0.38	0.707
Subendometrial 3D-PDA blood Flow				
VI	21.03 ± 16.39	17.54 ± 12.92	-0.86	0.387
FI	21.88 ± 2.92	20.73 ± 2.47	-1.75	0.080
VFI	4.89 ± 4.39	3.79 ± 3.40	-1.02	0.308
Endometrial contrast ultrasonography				
AT	16.09 ± 4.34	16.13 ± 3.77	-0.02	0.983
TTP	52.14 ± 143.39	29.37 ± 4.82	-0.00	0.997
PI	25.32 ± 4.56	24.16 ± 4.95	-1.01	0.312
AUC	2463.00 ± 627.01	2375.82 ± 585.69	-0.66	0.509
Subendometrial contrast ultrasonography				
AT	14.80 ± 4.08	14.73 ± 3.61	-0.07	0.945
TTP	28.92 ± 6.85	28.76 ± 5.89	0.00	1.000
PI	28.28 ± 4.89	27.03 ± 5.87	-0.71	0.476
AUC	3007.32 ± 789.03	2883.64 ± 745.30	-0.32	0.746
Endometrial blood flow grading, n(%)			10.65	0.005**
I	10 (22.73)	2 (4.76)		
II	24 (54.55)	18 (42.86)		
III	10 (22.73)	22 (52.38)		

EMT, Endometrial thickness; EV, Endometrial volume; VI, Vascularization index; FI, Flow index; VFI, Vascularization-flow index; AT, Arrival time; TTP, Time to peak; PI, Peak Intensity; AUC, Area under curve; -, Fisher exact. **P<0.01.

### 3.2 LASSO logistic regression analysis

**Figure 1:**
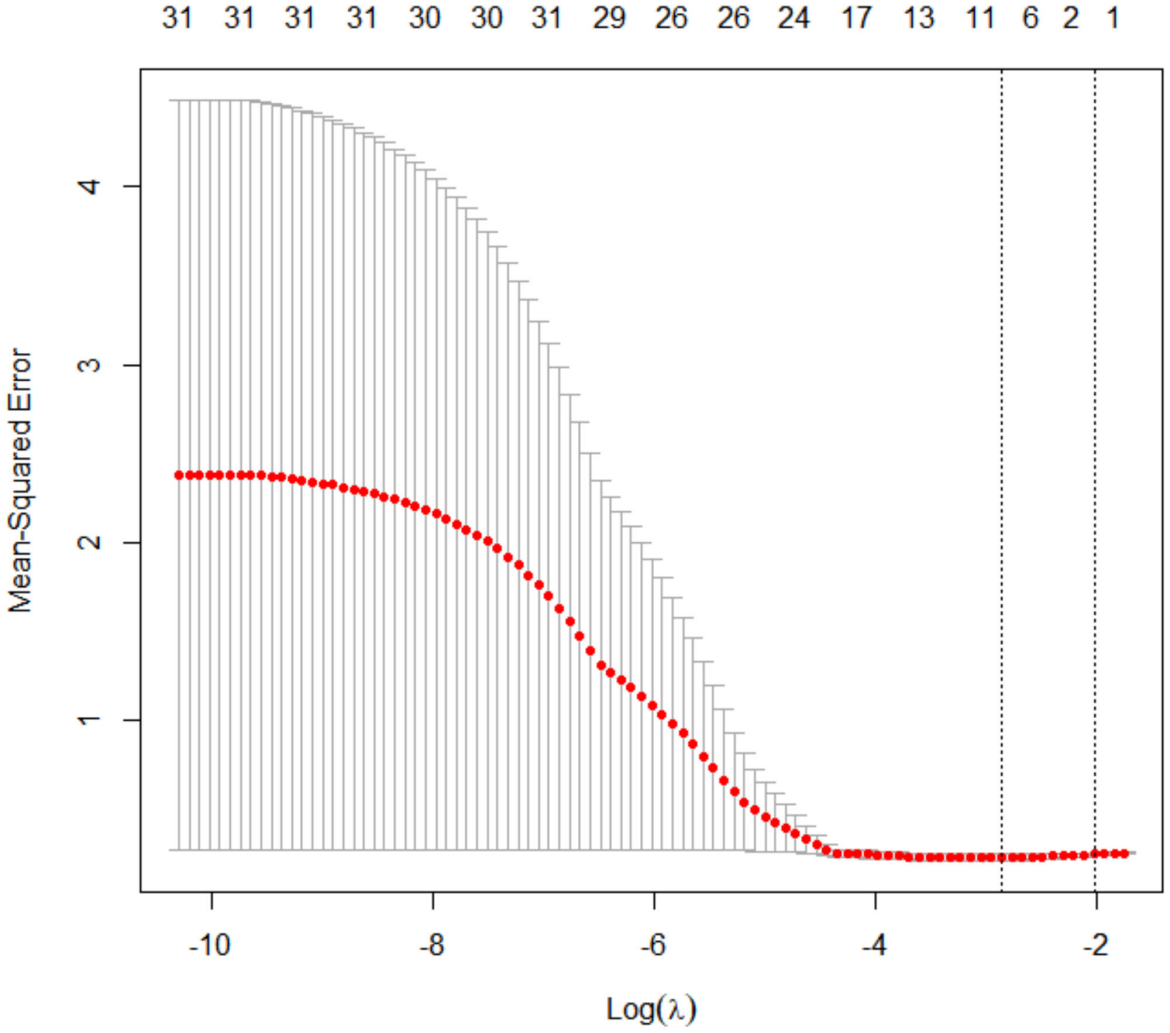
Lasso logistic regression cross-validation curve.

**Figure 2:**
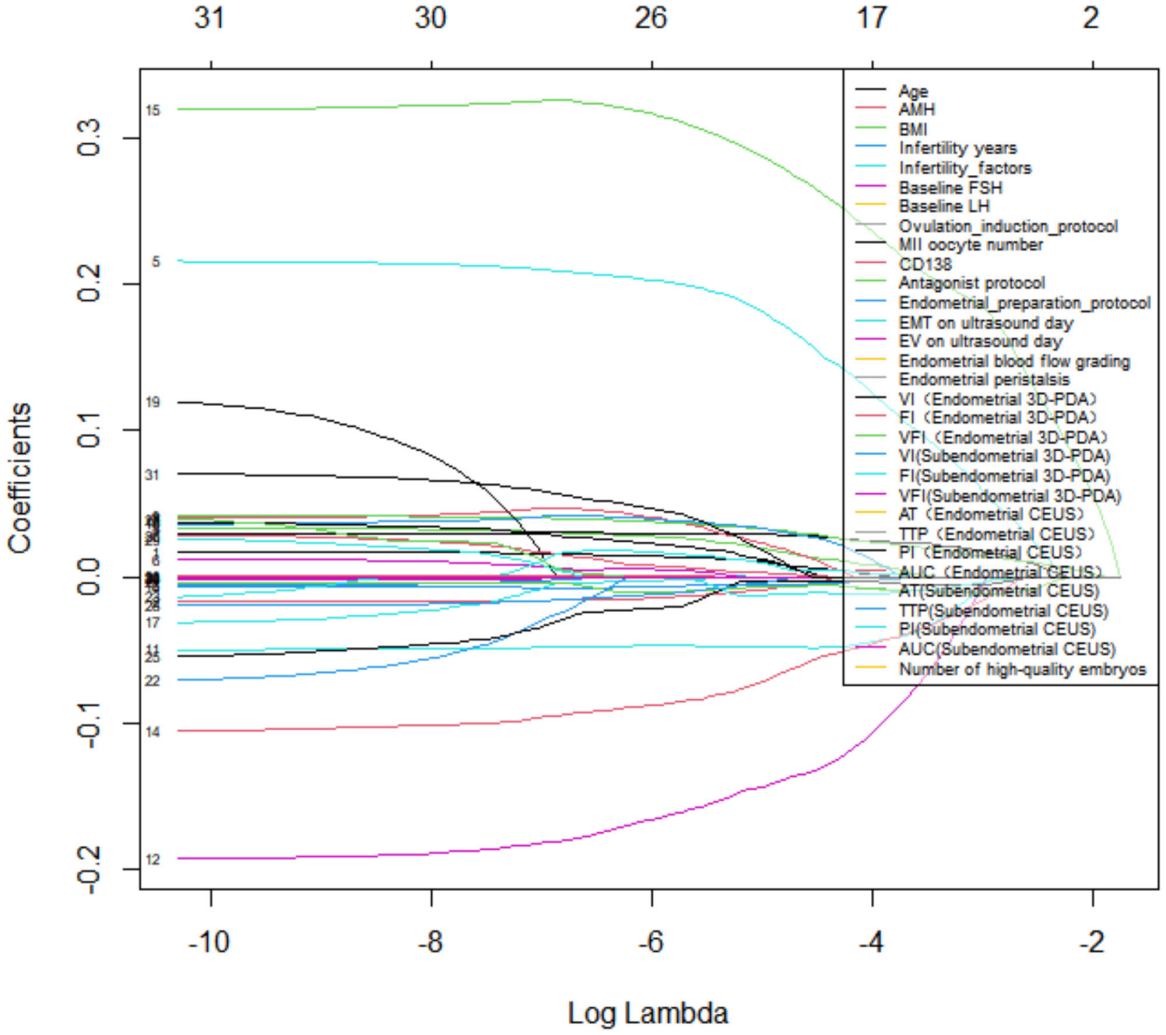
Lasso logistic regression analysis of correlates.

Using LASSO regression with 10-fold cross-validation (optimal λ = 0.576), we identified eight non-zero coefficient predictors: (1) infertility etiology, (2) baseline LH levels, (3) number of MII oocytes, (4) uterine cavity volume, (5) endometrial blood flow grading, (6) subendometrial flow index (FI) from 3D-PDA, and (7–8) endometrial and subendometrial PI from CEUS (**Figures 1**, **2**). There was no significant difference in the number of high-quality embryos transferred between groups (P = 0.169). However, since embryo quality is vital for IVF success, we included embryo factors in our statistical model.

### 3.3 Development of predictive models for ongoing pregnancy outcomes

**Figure 3:**
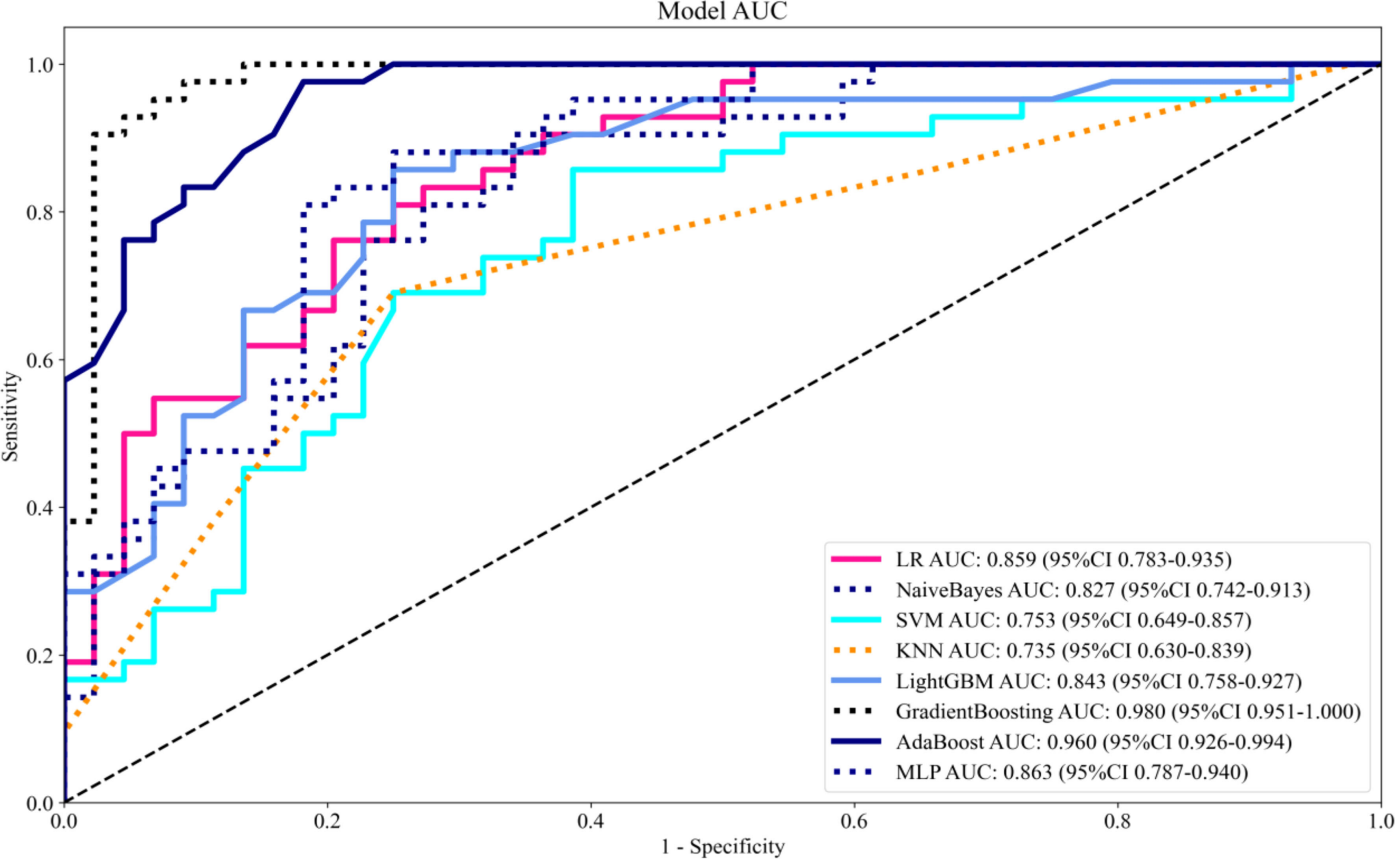
The AUC of onging prgenancy prediction model in different models.

**Figure 4:**
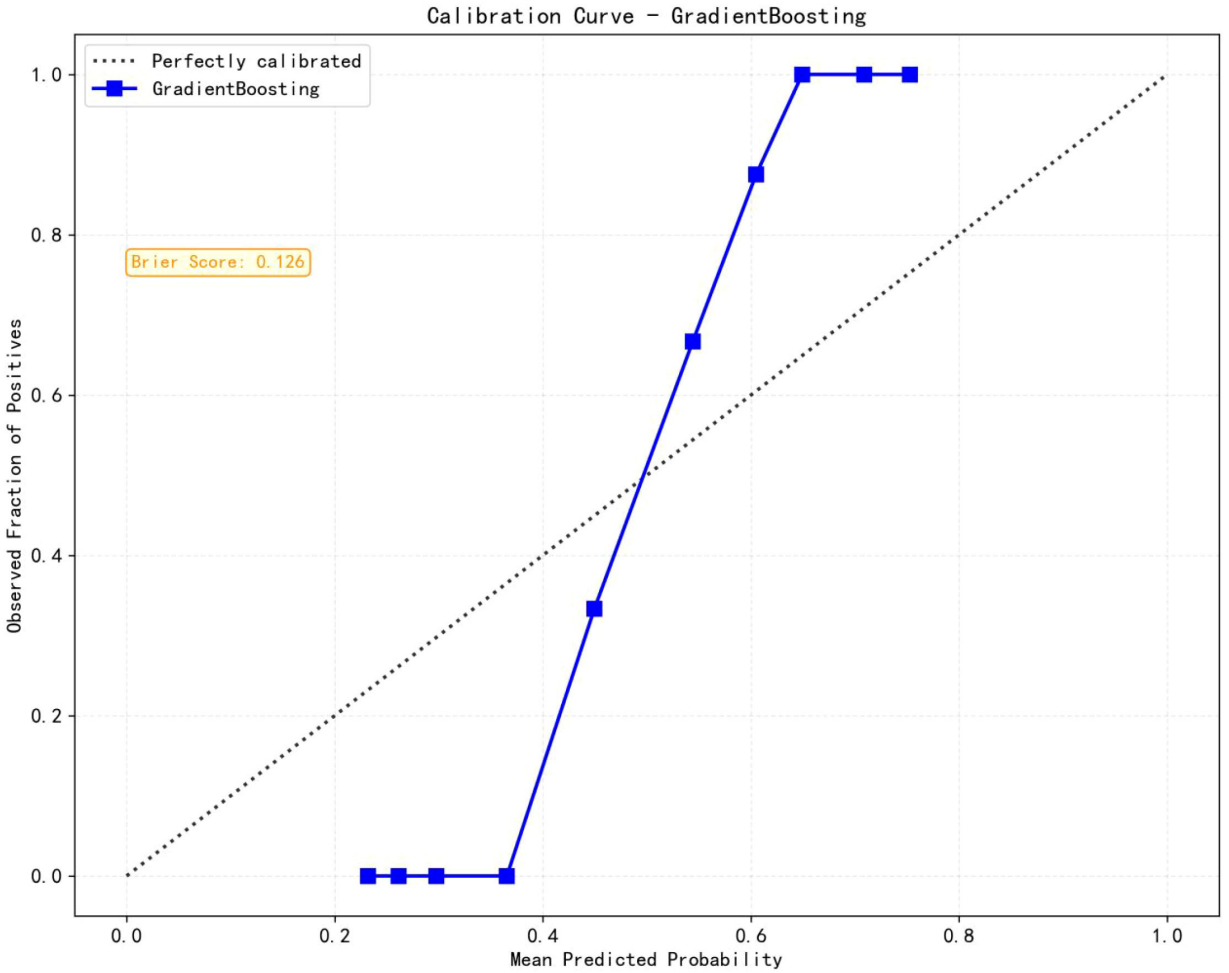
Calibration assessment of Gradient Boosting model.

Eight machine learning algorithms were trained using the selected predictive factors: Logistic Regression (AUC = 0.859, 95%CI 0.783-0.936), Support Vector Machine (AUC = 0.751, 95%CI 0.647-0.856), K-Nearest Neighbors (AUC = 0.722, 95%CI 0.615-0.829), Naive Bayes (AUC = 0.832, 95%CI 0.747-0.917), Gradient Boosting (AUC = 0.981, 95%CI 0.959-1.000), LightGBM (AUC = 0.843, 95%CI 0.758-0.927), AdaBoost (AUC = 0.960, 95%CI 0.926-0.994), and Multilayer Perceptron (AUC = 0.891, 95%CI 0.926-0.994). Comparative analysis revealed the Gradient Boosting model demonstrated optimal predictive performance (**Table 3**, **Figure 3**). The calibration assessment was performed on the optimal model, with the calibration plot illustrating the alignment between the model’s predicted probabilities and the observed probabilities of ongoing pregnancy. Furthermore, the Brier score served as a metric for evaluating the calibration curve’s performance, with lower Brier scores indicating greater model accuracy. The Gradient Boosting model attained a Brier score of 0.126, indicating robust predictive performance for ongoing pregnancy (see **Figure 4**).

**Table 3:** Performance evaluation of different models.

model_name	Accuracy	AUC	95% CI	SE	SPE	PPV	NPV	Precision	Recall	F1	Threshold
LR	0.802	0.859	0.783 - 0.936	0.929	0.682	0.736	0.909	0.736	0.929	0.821	0.386
NaiveBayes	0.791	0.832	0.747 - 0.917	0.857	0.727	0.750	0.842	0.750	0.857	0.800	0.303
SVM	0.721	0.751	0.647 - 0.856	0.857	0.591	0.667	0.812	0.667	0.857	0.750	0.452
KNN	0.721	0.722	0.615 - 0.829	0.738	0.705	0.705	0.738	0.705	0.738	0.721	0.600
LightGBM	0.802	0.843	0.758 - 0.927	0.857	0.750	0.766	0.846	0.766	0.857	0.809	0.492
Gradient Boosting	0.930	0.981	0.959 - 1.000	0.952	0.909	0.909	0.952	0.909	0.952	0.930	0.501
AdaBoost	0.895	0.960	0.926 - 0.994	0.976	0.818	0.837	0.973	0.837	0.976	0.901	0.491
MLP	0.826	0.891	0.826 - 0.957	0.881	0.773	0.787	0.872	0.787	0.881	0.831	0.487

SE, Sensitivity; SPE, Specificity; PPV, Positive Predictive Value; NPV, Negative Predictive Value.

**Figure 5:**
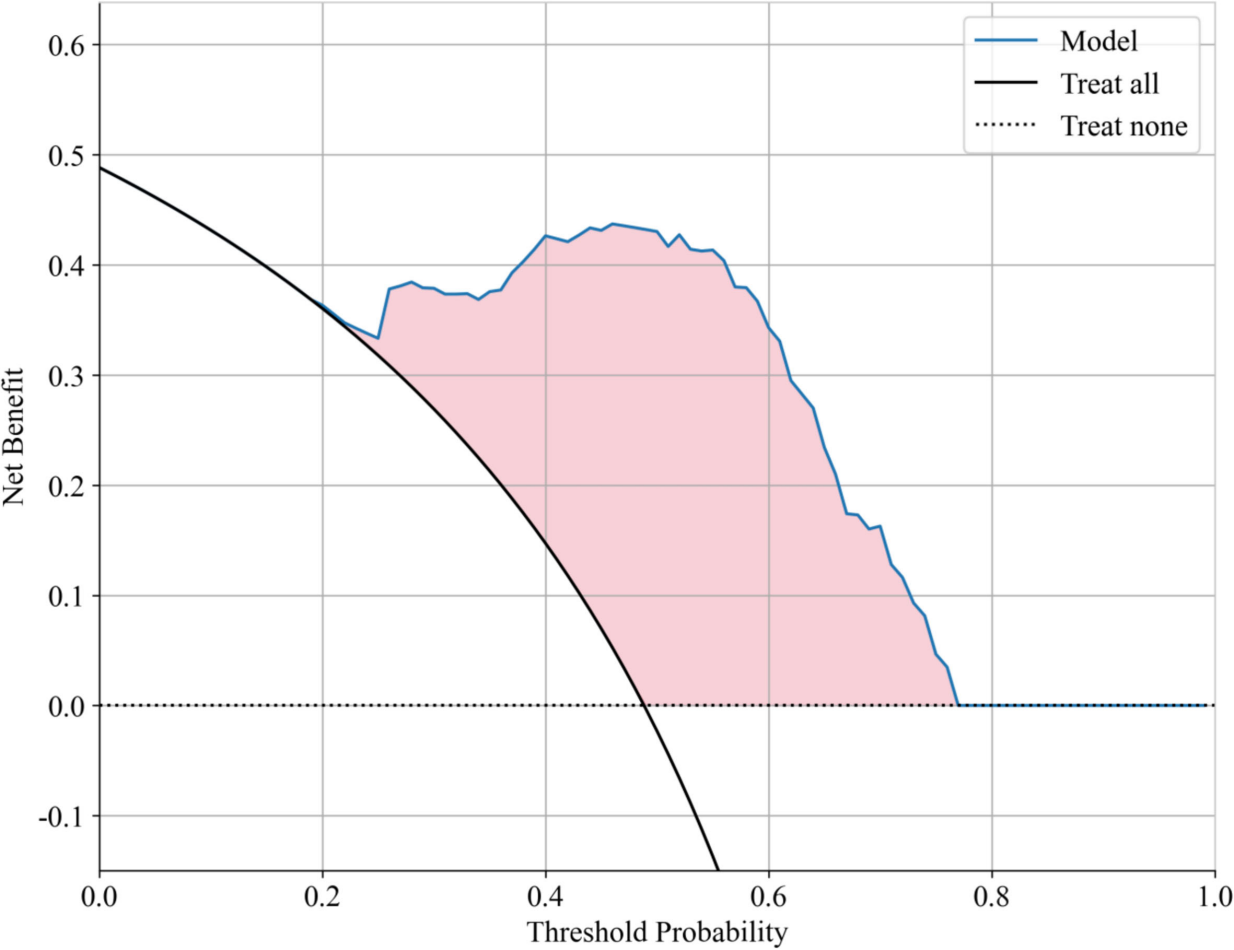
DAC curve for the ongoing pregnancy prediction model.

Decision curve analysis (DCA) further validated the clinical applicability of the Gradient Boosting model, showing significant net benefit across probability thresholds of 0.1-0.6 (**Figure 5**). The model achieved maximal clinical utility at thresholds of 0.2-0.4, demonstrating: 1) superior discrimination of moderate-risk patients compared to alternative approaches; 2) effective reduction of overtreatment in low-risk cases relative to universal treatment strategies; and 3) improved identification of high-risk patients versus conservative management approaches.

### 3.4 SHAP interpretation of Gradient Boosting model predictions

**Figure 6:**
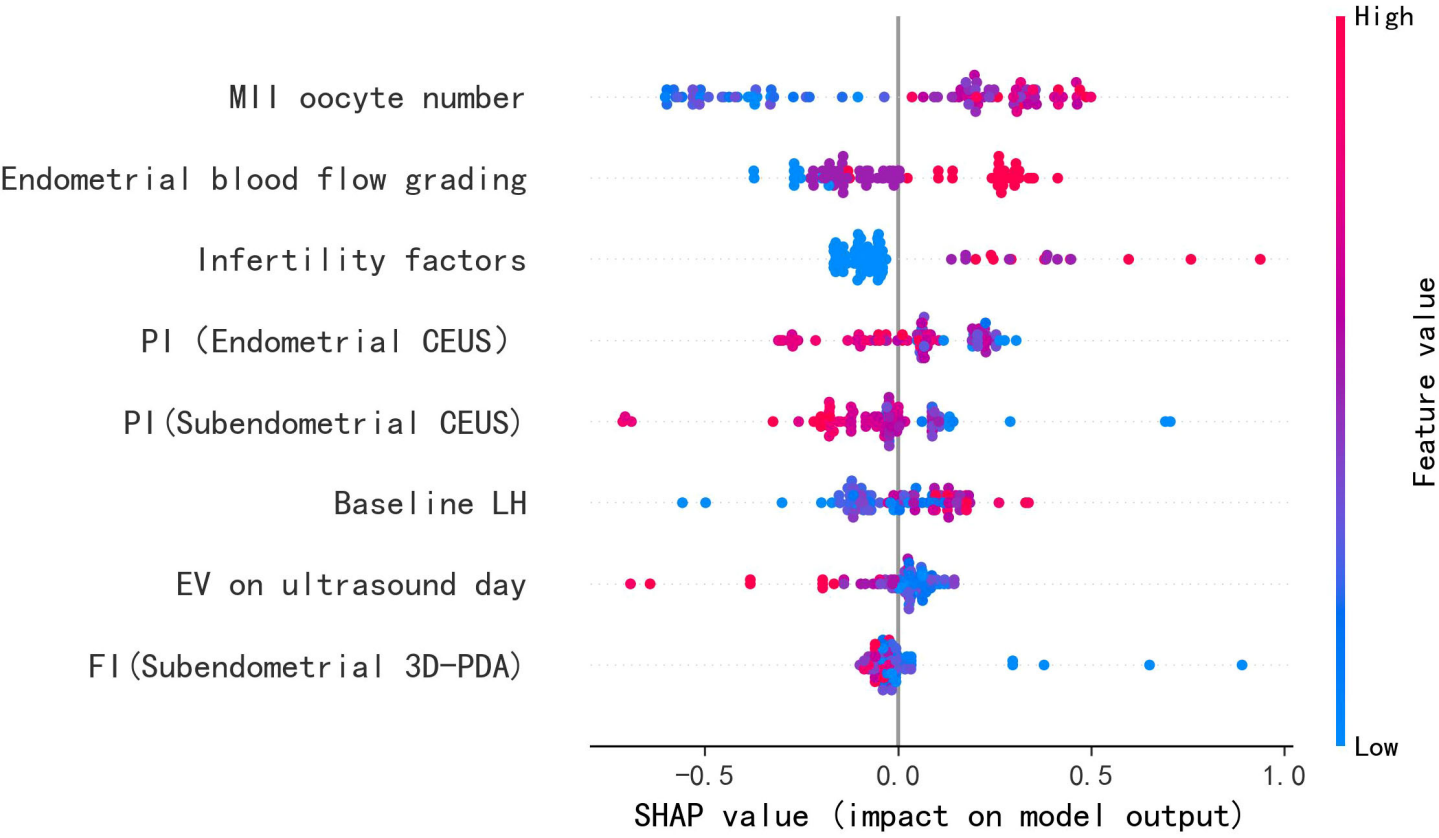
Correlation between each variable value and SHAP values.

The feature importance analysis using SHapley Additive exPlanations (SHAP) is presented in **Figure 6** through violin plots, demonstrating the magnitude and direction of each variable’s contribution to the model predictions. Higher absolute SHAP values correspond to greater predictive importance, with red and blue data points representing high and low feature values, respectively. Key positive predictors of ongoing pregnancy included: (1) higher numbers of MII oocytes retrieved, (2) improved endometrial blood flow grading, and (3) elevated baseline LH levels. Conversely, reduced pregnancy likelihood was associated with increased values of: (1) endometrial and subendometrial PI, (2) FI, and (3) uterine cavity volume.

**Figure 7:**
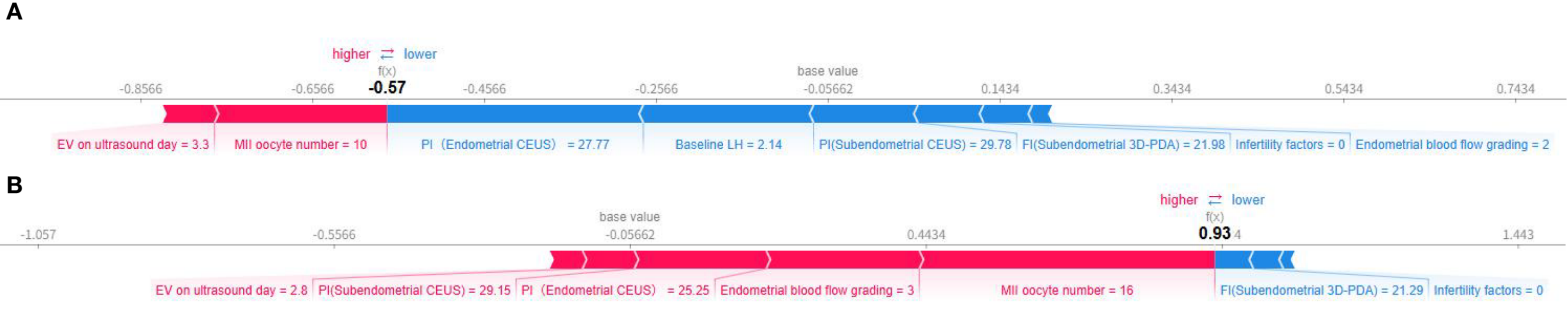
Individual force diagram of gradient boosting prediction model. **(A)** Non-pregnant, **(B)** Ongoing pregnancy.

Individual prediction explanations are visualized through force plots (**Figures 7A, B**), where the horizontal axis represents the cumulative SHAP value driving the prediction from the baseline output. For pregnancy failures (**Figure 7A**), the dominant contributing features were enlarged uterine cavity volume and suboptimal MII oocyte yield. Successful pregnancy predictions (**Figure 7B**) were primarily influenced by adequate MII oocyte numbers, favorable endometrial blood flow patterns, and optimal PI values. The directionality of each feature’s impact is indicated by arrow orientation (positive/negative) and color intensity (magnitude of contribution).

### 3.5 Internal validation and efficacy calculation of Gradient Boosting model

**Figure 8:**
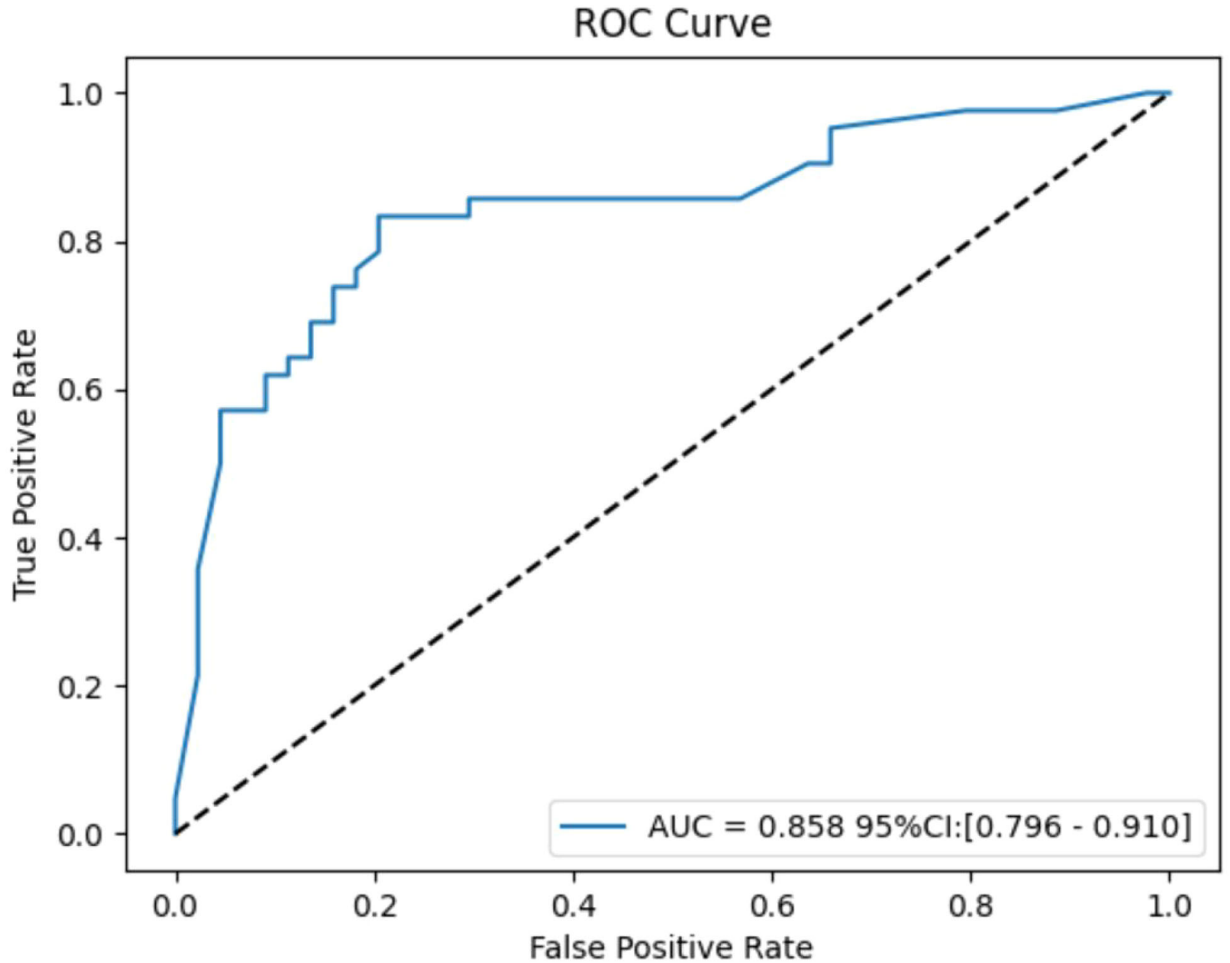
Internal validation of Gradient Boosting model.

Bootstrap analysis with 1000 resamples was used for internal validation, yielding an average AUC of 0.858 (95% CI: 0.796-0.910). The effect size, calculated using the Hanley & McNeil method, was 2.963, and the event per variable (EPV) ratio was 4.7:1. While the high AUC and effect size show strong discriminative ability, the low EPV ratio suggests potential overfitting. Internal validation efforts were made to address this concern (**Figure 8**).

## 4 Discussion

Successful embryo implantation represents a critical determinant of pregnancy achievement in IVF-ET, with both embryo quality and quantity serving as pivotal factors. Our findings corroborate previous studies demonstrating that diminished MII oocyte yield significantly predicts implantation failure, likely through limiting the availability of genetically competent gametes for fertilization. Simultaneously, it decreases transplantable embryos, with embryo quality being crucial for IVF success (12). The observed association between elevated baseline LH levels and improved reproductive outcomes may reflect enhanced follicular recruitment and subsequent oocyte quality, consistent with LH’s established roles in promoting embryo development and maintaining luteal function.

The cyclical endometrial changes during the luteal phase (days 21-22) create an optimal microenvironment for implantation through coordinated hormonal actions. Progesterone and estrogen synergistically induce characteristic morphological changes including stromal edema, glandular coiling, and vascular proliferation. Notably, the spiral arteries undergo marked dilatation and tortuosity to accommodate the increased hemodynamic demands post-implantation. Our data support existing literature demonstrating superior pregnancy rates with favorable endometrial perfusion characteristics (13). Specifically, we observed significantly greater subendometrial vascularity (Type II/III patterns) in conception cycles (94.3% vs 91.8%, p<0.05) (14), aligning with Sun et al.’s findings (7). However, the moderate predictive accuracy (AUC = 0.567) of blood flow patterns alone underscores the need for multimodal assessment. The transition from Type II/III to Type I vascular patterns following oocyte retrieval, as described by Guo et al. (15), may explain the observed association between peri-retrieval blood flow impairment and early pregnancy loss (21.74% vs 9.23%). While our study confirms the prognostic value of endometrial blood flow grading, the current lack of standardized classification systems and limited prospective validation studies continue to generate controversy regarding its clinical utility.

The spiral arteries’ low-velocity flow and complex structure can lead to a low number/area ratio and unstable perfusion timing, making it difficult to detect perfusion changes in the endometrium using 2D ultrasound. While 3D-PDA is more sensitive to low-velocity blood flow than traditional Doppler methods, its clinical value for assessing endometrial vasculature is still debated (16, 17). CEUS, a significant advancement in ultrasound imaging, is now widely used in clinical practice for its superior ability to evaluate blood flow perfusion. Meta-analytic data (18) and nomogram studies (19–21) consistently identify FI as the most robust predictor among vascular parameters, though our results suggest subendometrial indices may show inverse relationships with pregnancy outcomes in certain populations. Several factors may account for these discrepancies: First, the restricted sample size may have limited power. Second, protocol-driven anticoagulant administration could have modified natural hemodynamic patterns. Third, single-timepoint assessments fail to capture the dynamic vascular changes occurring throughout the menstrual cycle (22). The observed values in our pregnant cohort, while lower than non-pregnant controls, exceeded established diagnostic thresholds (21, 23), possibly reflecting progesterone-mediated vascular effects in ART cycles.

There is currently no consensus on a standard for multimodal ultrasound assessment of endometrial receptivity. Developing a scoring system based on ultrasound characteristics may involve subjectivity, impacting its clinical utility. The first-trimester pregnancy prediction model using three-dimensional ultrasound parameters shows moderate diagnostic performance (AUC = 0.639), and the logistic regression model with clinical parameters and endometrial elasticity indicators also has limited accuracy (8–10). Our study extracts key features from 3D-PDA and CEUS parameters using a data-driven approach to reduce subjective bias. The Gradient Boosting model, optimized and evaluated, achieved the highest performance (AUC = 0.981). We use the SHAP method to assess feature importance, enhancing model interpretability. Practically, our model improves endometrial receptivity assessment, aiding clinical treatment planning. For example, in assisted reproductive technology, it helps determine the best embryo transfer timing, boosting transplantation success rates.

Regarding safety, sulfur hexafluoride microbubble contrast agents demonstrate excellent safety profiles with no evidence of teratogenicity even at supratherapeutic doses (24). The isolated febrile episode in our series appeared unrelated to contrast administration. While preliminary results are encouraging, larger randomized controlled trials remain necessary to establish definitive safety guidelines and validate the clinical efficacy of contrast-enhanced techniques in reproductive medicine.

This study has limitations, including its single-center, exploratory nature and small sample size, which may affect statistical power and limit generalizability. Future research should involve large-scale, multicenter trials for validation. The study’s primary outcome was sustained pregnancy, lacking final live birth rate data, which is the ultimate efficacy measure in assisted reproductive technology. As of follow-up, some patients hadn’t reached live birth, so outcomes during mid-to-late pregnancy weren’t covered. Further follow-up and analysis of factors affecting live birth are needed. Internal validation showed potential overfitting due to a low EPV ratio, indicating the need for more data collection. No reproducibility analysis was performed, which is important when considering use of a method in clinical practice. A better design including independent acquisition and blinded analysis should be considered in future work.

## 5 Conclusion

Assessing endometrial receptivity using multimodal ultrasound, including contrast-enhanced techniques, is crucial for predicting IVF-embryo transfer success. Our model shows that combining endometrial blood flow and mature oocyte count improves prediction accuracy. Clinically, this can optimize ovarian stimulation and suggest blood perfusion therapy for patients with poor vascular parameters. Future studies should confirm these results in larger human cohorts and explore more biomarkers to enhance the model. This ultrasound-based predictive model, developed with multimodal ultrasound parameters, advances precision medicine by providing a refined, noninvasive method for assessing endometrial receptivity and guiding personalized clinical interventions.

## Data Availability

The original contributions presented in the study are included in the article/supplementary material. Further inquiries can be directed to the corresponding author.
