# Correction: Analysis of ultrasound parameters influencing endometrial receptivity and a pregnancy outcomes predictive model for patients undergoing *in vitro* fertilization and embryo transfer: a prospective study

**DOI:** 10.3389/fendo.2026.1840394

**Published:** 2026-04-20

**Authors:** Chunlian Wang, Jiao Lin, Xingping Zhao, Ni Liu, Pengzi Sun, Jiaoli Yang, Xue Zhou

**Affiliations:** 1 Obstetrics and Gynecology Ultrasound Department, Xiangtan Central Hospital, Affiliated Hospital of Hunan University, Xiangtan, Hunan, China; 2 Reproductive Center, Xiangtan Central Hospital, Affiliated Hospital of Hunan University, Xiangtan, Hunan, China; 3 Reproductive Center, Third Xiangya Hospital, Affiliated Hospital of Zhongnan University, Changsha, Hunan, China

**Keywords:** ultrasound parameters, endometrial contrast-enhanced ultrasound, IVF-ET, ongoing pregnancy, prediction model

There was a mistake in the **Abstract/Introduction/Conclusion** section, an incorrect words was used.

A correction has been made to the sections **Abstract**, **Methods**, Page 1; **Introduction**, third paragraph, Page 2; and **Conclusion**, Page 11.

**Abstract** section, “**Methods**: The prospective cohort study included 86 patients treated at the Reproductive Center of Xiangtan Central Hospital from May to December 2024. Participants underwent multimodal ultrasound evaluation one day before embryo transfer. The study analyzed endometrial morphology, blood flow parameters, as well as three-dimensional power Doppler angiography(3D-PDA), and endometrial contrast-enhanced ultrasound (CEUS) indicators. Multimodal ultrasound parameters were used to establish a predictive model for sustained pregnancy.”

**Introduction** section, third paragraph: “An optimal blood supply to the endometrium is essential for embryo implantation. During early implantation, endometrial vascular permeability increases, causing significant changes in the microvasculature of endometrium and subendometrial region. As the primary decidual zone develops, capillaries near the embryo close while those adjacent to the decidual zone dilate. Impaired endometrial angiogenesis may lead to recurrent implantation failure andpregnancy loss. Increased vascular permeability aids in delivering growth factors and cytokines to the implantation site (11). CEUS provides real-time visualization of tissue microcirculation, while 3D-PDA effectively detects low-velocity blood flow with outangle limitations. 3D-PDA captures blood flow signals from all directions, enabling comprehensive volume analysis through computer reconstruction. This study seeks to address this gap by developing a more precise evaluation model that incorporates a comprehensive array of ultrasound indicators related to ER, encompassing physiological and morphological characteristics, blood flow dynamics, and advanced imaging parameters such as 3D-PDA and CEUS.”

“**Conclusion**: Assessing endometrial receptivity using multimodal ultrasound, including contrast-enhanced techniques, is crucial for predicting IVF-embryo transfer success. Our model shows that combining endometrial blood flow and mature oocyte count improves prediction accuracy. Clinically, this can optimize ovarian stimulation and suggest blood perfusion therapy for patients with poor vascular parameters. Future studies should confirm these results in larger human cohorts and explore more biomarkers to enhance the model. This ultrasound-based predictive model, developed with multimodal ultrasound parameters, advances precision medicine by providing a refined, noninvasive method for assessing endometrial receptivity and guiding personalized clinical interventions.”

The original version of this article has been updated.

